# L’amputation de Chase dans un mélanome de l’index: à propos d’un cas

**DOI:** 10.11604/pamj.2017.27.188.8512

**Published:** 2017-07-11

**Authors:** Tarik Madani, Mohamed Amine Karabila, Younes Mhammdi, Mohammed Kharmaz, Mohamed El Ouadghiri, Abdou Lahlou, Ahmed El Bardouni, Mustapha Mahfoud, Mohamed Saleh Berrada

**Affiliations:** 1 Service de Chirurgie Orthopédique et Traumatologique du Centre Hospitalier Universitaire de Rabat, Maroc

**Keywords:** Amputation, index, melanome, Amputation, index, melanome

## Abstract

Nous rapportons le cas d'une patiente de 40 ans, adressée par le service de dermatologie de l'hôpital Ibn-Sina de Rabat pour amputation de l'index suite à un mélanome diagnostiqué sur une biopsie. L'amputation a été réalisée selon la technique de Chase. Le résultat esthétique et fonctionnel était très satisfaisant après six mois de l'intervention.

## Abstract (en)

We report the case of a 40-year old patient referred by the Depatment of Dermatology at the Ibn Sina Hospital in Rabat for amputation of the index finger as a result of a melanoma diagnosed by biopsy. The amputation was performed according to Chase’s method. Aesthetic and functional outcome was very good six months following surgical treatment.

## Introduction

L'intervention de Chase répand aux objectifs de traitement par indexalisation du médius qui va assurer la prise pollicidigitale [1]; conserver la force; et respecter l'aspect esthétique de la main. Nous rapportons le cas d'une patiente de 40 ans, adressée par le service de dermatologie de l'hôpital Ibn-Sina de Rabat pour amputation de l'index suite à un mélanome diagnostiqué sur une biopsie. L'amputation a été réalisée selon l'intervention de Chase. Le résultat esthétique et fonctionnel était très satisfaisant après six mois de l'intervention.

## Patient et observation

**Figure 1:**
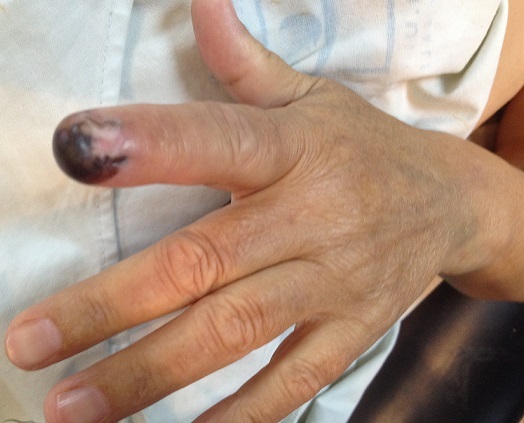
Aspect noirâtre de l’extrémité distale de l’index avec déformation de l’ongle

**Figure 2:**
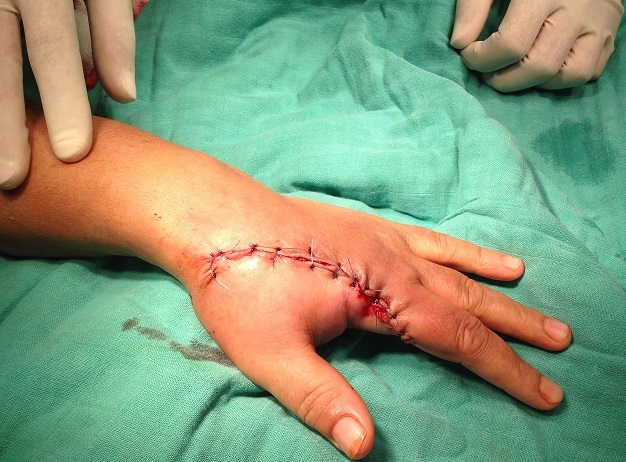
Indexalisation du médius et conservation du galbe de la première commissure

Il s'agit d'une patiente âgée de 40 ans, droitière, femme au foyer, admise pour une tuméfaction douloureuse au niveau de l'index gauche qui remonte à deux ans augmentant progressivement de volume. L'examen clinique trouve un aspect noirâtre au niveau de P3 du 2^ème^ doigt gauche prenant l'ongle et les parties molles (Figure 1), l'examen des aires ganglionnaires est normal. La radiographie objective une image lytique de la troisième phalange de l'index qui s'étant à l'articulation IPD. La biopsie est revenue en faveur d'un mélanome. La patiente a bénéficié d'une amputation selon la technique de Chase qui consiste en une amputation à la base du deuxième métacarpien ce qui permet l'indexalisation du médius (Figure 2). La patiente a bénéficié d'une rééducation précoce. Le contrôle après 6 mois objective un bon résultat esthétique et fonctionnel de la main avec une bonne ouverture de la commissure pouce-médius, indexalisation du médius avec satisfaction de la patiente de l'aspect esthétique et fonctionnelle de sa main.

## Discussion

L'index est le doigt le plus utilisé après le pouce. Il s'agit d'un élément essentiel de la prise pollicidigitale et d'un élément de stabilisation dans la préhension digitopalmaire globale. Toute amputation, si petite soit-elle, altère la préhension [2]. L'amputation de Chase: c'est une amputation à la base du deuxième métacarpien ce qui permet l'indexalisation du médius elle a l'avantages d'assurer une ouverture maximale de la commissure pouce-médius [2]. L'indexalisation du médius conduit à une meilleure intégration et utilité du doigt [3]. Protocole opératoire [4]: Temps cutané: l'incision est le plus souvent dorsale afin d'éviter une cicatrice palmaire potentiellement gênante, elle circonscrit en « raquette » la base de l'index; Temps tendineux et osseux: par voie dorsale, l'appareil extenseur est sectionné. Le tendon extensor indicis est sectionné en amont de l'articulation métacarpophalangienne, puis transféré sur le tendon extensor digitorum destiné au médius par une suture latérolatérale. L'adductor pollicis est désinséré du deuxième métacarpien et la section osseuse de la base est oblique en bas et en dehors, en conservant l'articulation carpométacarpienne. Les tendons fléchisseurs sont sectionnés en proximal, poignet en flexion. Le tendon terminal du premier interosseus dorsale est suturé au tendon du deuxième interosseus. Ce transfert permet une inclinaison radiale efficace dans les prises pouce-médius ainsi qu'une meilleure force; Temps vasculonerveux: les nerfs collatéraux, disséqués en proximal, sont sectionnés haut dans la paume. Pour certains, les nerfs collatéraux peuvent, après section, être enfouis dans le premier interosseus dorsale. Grace a cette technique la dextérité de la main est grandement améliorée et son aspect est plus esthétique [5]. L'inconvénient rapporté dans la littérature est la diminution de la force globale de la main, surtout en pronation par la diminution de la largeur de la main [6].

## Conclusion

La chirurgie est le traitement de référence et souvent le seul traitement nécessaire des cancers cutanés à condition que le geste soit carcinologique. Cependant dans les tumeurs des doigts il faut aussi prendre en considération la fonction de la main, le principe est de ne pas conserver un niveau qui n'est pas fonctionnellement meilleur que celui que l'on aurait obtenu en raccourcissant l'os; pour donner une fonction correcte de la main.

## Conflits d’intérêts

Les auteurs ne déclarent aucun conflit d'intérêt.
